# The development of instruments to measure the work disability assessment behaviour of insurance physicians

**DOI:** 10.1186/1471-2458-11-1

**Published:** 2011-01-03

**Authors:** Romy Steenbeek, Antonius JM Schellart, Henny Mulders, Johannes R Anema, Herman Kroneman, Jan Besseling

**Affiliations:** 1 TNO Work and Employment, PO Box 718, 2130 AS Hoofddorp, the Netherlands; 2 VU University Medical Center, Department of Public and Occupational Health, EMGO Institute for Health and Care Research, Amsterdam, the Netherlands; 3 UWV, Employee Benefits Insurance Authority, Amsterdam, the Netherlands; 4 Research Center for Insurance Medicine, AMC-UWV-VUmc, Amsterdam, the Netherlands

## Abstract

**Background:**

Variation in assessments is a universal given, and work disability assessments by insurance physicians are no exception. Little is known about the considerations and views of insurance physicians that may partly explain such variation. On the basis of the Attitude - Social norm - self Efficacy (ASE) model, we have developed measurement instruments for assessment behaviour and its determinants.

**Methods:**

Based on theory and interviews with insurance physicians the questionnaire included blocks of items concerning background variables, intentions, attitudes, social norms, self-efficacy, knowledge, barriers and behaviour of the insurance physicians in relation to work disability assessment issues. The responses of 231 insurance physicians were suitable for further analysis. Factor analysis and reliability analysis were used to form scale variables and homogeneity analysis was used to form dimension variables. Thus, we included 169 of the 177 original items.

**Results:**

Factor analysis and reliability analysis yielded 29 scales with sufficient reliability. Homogeneity analysis yielded 19 dimensions. Scales and dimensions fitted with the concepts of the ASE model. We slightly modified the ASE model by dividing behaviour into two blocks: behaviour that reflects the assessment process and behaviour that reflects assessment behaviour.

The picture that emerged from the descriptive results was of a group of physicians who were motivated in their job and positive about the Dutch social security system in general. However, only half of them had a positive opinion about the Dutch Work and Income (Capacity for Work) Act (WIA). They also reported serious barriers, the most common of which was work pressure. Finally, 73% of the insurance physicians described the majority of their cases as 'difficult'.

**Conclusions:**

The scales and dimensions developed appear to be valid and offer a promising basis for future research. The results suggest that the underlying ASE model, in modified form, is suitable for describing the assessment behaviour of insurance physicians and the determinants of this behaviour. The next step in this line of research should be to validate the model using structural equation modelling. Finally, the predictive value should be tested in relation to outcome measurements of work disability assessments.

## Background

Variation in assessments by professionals is a well-known phenomenon which occurs in cases where assessments are carried out by several raters and in various disciplines. In the case of physicians, variations occur in the diagnosis and treatment of patients. A degree of uncertainty is inherent in the profession and it is very easy to reach different conclusions in comparable cases [1,2]. Inter-doctor variation in diagnosis and/or treatment is found in different medical disciplines [3-7]. Specific research into variation among GPs shows variation in diagnosis [8,9], request for interventions [8,10-12], treatment [13,14] and rate of referral to specialists [15]. Literature on insurance physicians is less extensive but variation in the assessment of functional capacity for work exists [16-18]. In this study we concentrate on how insurance physicians assess workers' claims to compensation for loss of work capacity. Insurance physicians have to judge a claim and in doing so base themselves on the available information (file) and information provided by the "client", i.e. the patient who claims a work disability benefit, and others. The outcome of the assessment, i.e. the functional capacity for work, is variable. An important aspect of this assessment is the client's medical situation in the context of the current social security legislation. In making the assessment the insurance physician must therefore deal with the characteristics of both the legal and the medical decision-making process. Assessing work disability is therefore a complex and specialised process that also gives rise to variation in outcomes. Ydreborg and Ekberg [16] found variations in the extent to which applicants for disability pension were rejected in practice. Spanjer et al. [17] evaluated inter-rater reliability between insurance physicians in respect of physical disability and mental disability assessment as reasonable to good. However, inter-rater reliability in respect of the assessment of the number of hours clients could function daily was low. Spanjer et al. [18] found a significant difference in various scores on assessed work limitation items by insurance physicians.

The outcome of work disability assessments by insurance physicians can be seen as the result of behaviour influenced by various factors, including behavioural determinants of the physicians in relation to the intended object of their assessment. Little is known of what considerations and views of insurance physicians may partly account for variation in the outcome of assessments.

## Conceptualisation

It is evident from the above that variation generally occurs in assessments by physicians. Research shows that this variation is connected with, among other things, certain personal characteristics [13,19,20] and behavioural characteristics, such as personal style or attitude [11,13,21]. Systematic variation between insurance physicians in the outcomes of assessments (i.e. grant or reject the claim) can be regarded as the result of assessment behaviour which - in addition to other factors - is determined in part by the attitude which the insurance physician has towards the intended purpose of his assessment. Thus, assessment behaviour is defined as all behaviour that may influence the outcome of the assessment, including the collection and evaluation of information about the client.

A theory of the relationship between attitudes and behaviour was elaborated in the literature of the period 1970-1980 by Fishbein and Ajzen in their 'Theory of Reasoned Action (TRA)' [22,23]. In the 1980 s this theory was taken a step further by Azjen [24] in his 'Theory of Planned Behaviour (TPB)'. He added 'perceived behavioural control' (self-efficacy) as a factor that moderates behaviour. In the second half of the 1980 s Azjen's model was supplemented by elements from the 'Social Cognitive Theory (SCT)' of Bandura [25] in the so-called ASE model [26]. ASE is a model that has general scientific acceptance and explains behaviour by linking attitude, social influence and self-efficacy with behaviour and behavioural intention [27]. In addition to the three determinants of intention and behaviour, intermediary factors such as 'knowledge' and 'barriers' can play a role. TPB and the ASE model are used in the Netherlands to explain, among other things, the behaviour of physicians [28] and patients in an occupational health context [29-32] and the health behaviour of individuals who belong to a particular target group [33,34]. On the basis of TPB research Croon & Langius [29] have studied attitudes and working styles (behavioural intentions) among insurance physicians who assessed employees' sick leave not exceeding one year. The present survey takes the ASE model as the basis for possible explanations of the behaviour of insurance physicians in assessing work disability after sick leave lasting one year (Disability Insurance Act -WAO), two years (Work and Income (Capacity for Work) Act - WIA) or more years (Adapted Reassessment Act - HERBO, see next paragraph).

In the Netherlands, if you are partially or fully incapable of working after two years of illness, you may be eligible to receive a benefit under the Work and Income (Capacity for Work) Act (WIA). The WIA succeeded the Disability Insurance Act (WAO) in January 2006. The WAO was not repealed by the WIA, but now applies only to those who were already receiving a WAO benefit on 1 January 2006. The Adapted Re-assessment Act (HERBO) was introduced in August 2004 for the reassessment of WAO benefits clients, i.e. the claimants (< 50 years), on the basis of new, stricter criteria that put the emphasis on the client's residual functional capacities. These stricter assessment rules under HERBO also apply to the WIA. Young disabled people may be eligible to receive a benefit under the Invalidity Insurance (Young Disabled Persons) Act (Wajong). The WAO and WIA differ in the time of assessment. The WAO provides for assessments after one year of illness, whereas the WIA provides for assessments after two years of illness. In this study, we will only use disability assessment outcomes under WAO, HERBO and WIA.

**Figure 1:**
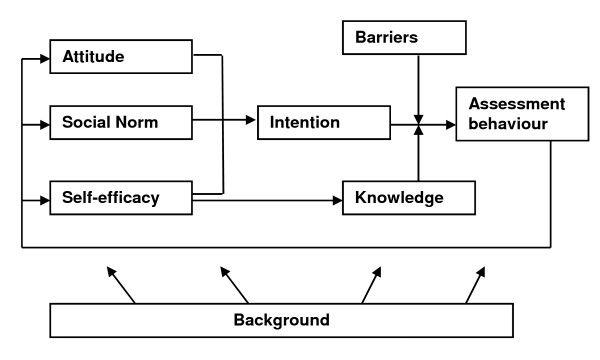
**The ASE model**.

For parts of the ASE model (see figure 1) research has shown that there is a correlation with variation in assessments by physicians. Research among GPs shows varying conclusions as regards attitude. Taylor concluded as long ago as 1977 in a review [13] that variation in prescribing behaviour was associated with the personal attitudes of GPs. In addition, the results of Grytten and Sorensen [11] indicate that practice style reflects a deeply rooted behaviour with respect to how to practise medicine. However, Tellnes et al. [35] and Thies-Zajong et al. [36] found no association between doctors' attitudes and some measures of variation. Social norms also appear to play a role. The degree to which insurance physicians are required to achieve a certain level of production and the pressure exerted on them to achieve the targets differ from office to office [37-39]. No literature was found on the relationship between self-efficacy and assessments by physicians. Research among GPs shows that a higher workload (operationalised as list size) functions as a barrier; it leads to more referrals to specialists [15]. A lack of sufficient knowledge and information can also serve as a barrier. Davis et al. [40] found that diagnostic uncertainty in GPs was associated with higher rates of investigation and follow-up. Finally, client or patient characteristics such as gender [20], unemployment and age [16] or the type of patient visit [4] were found to be associated with variations in outcome.

## Applying the conceptualisation

Below we describe the factors in respect of which an association has been found with variations in assessments by physicians for each concept from the ASE model and which have been included in our model. Our search for literature on the specific relationship between certain factors and variation in assessments revealed that few evidence-based and peer-reviewed articles exist. This is why we also used 'grey literature'. Literature on insurance physician's behaviour is scarce. That is why we added research on GPs, because in many countries the GP is the physician issuing sickness certificates.

### Background variables

The gender of the physician appears to influence assessment behaviour. Female insurance physicians more frequently restrict the number of hours a client can work as part of their assessment [40]. Court [41] showed this with specific regard to Dutch insurance physicians: male insurance physicians conclude less frequently than female insurance physicians that clients are not able to work.

Age is closely connected with experience: the older the doctor the more experience he has. Both factors influence assessment behaviour. Physicians with more experience in family medicine issued more sickness certificates [42] and the duration of episodes of sickness certification was longer for patients of older doctors [35]. In the case of insurance physicians, greater experience is associated with greater optimism about the patient's return to work [43], better quality assessment in the case of mental complaints [44], more frequent allowance of a reduction in working hours [45], a tendency to assess suitability for work as higher in the event of reassessments [43] and a shift in thinking from the 'seriousness of the complaint' to the 'extent to which the complaints influence daily functioning' [46].

Training [35,42] and specialisation [19] are also connected with the assessment behaviour of physicians. Physicians who were trained in social insurance medicine as undergraduates issued more sickness certificates [42]. The duration of episodes of sickness certification was shorter in patients of doctors with postgraduate training [35].

The number of working hours is also a factor that influences sickness certification: physicians working part time issued more sickness certificates than physicians working full time [42].

Assessments also vary according to locations or region: the variation between these units has been shown to be greater than within them [12,16,47,48].

### Attitude

Taylor concluded as long ago as 1977 [13] that more desirable prescribing patterns by GPs were associated with a more psychosocial orientation towards medical care. In case of insurance physicians, desirable behaviour could be associated with perceived justness of the social security system.

The attitude towards the perceived quality of the assessment may also influence it. Insurance physicians are dissatisfied about the scope for development and refresher training and complain about a lack of clear instructions in the legislation, regulations and instruments [49,50]. Views on quality are also influenced by increased production pressure and the feeling of having too little time for the assessments [41].

Finally, the physician's attitude towards the personal needs and circumstances of the client (including his recovery) can influence the assessment. Physicians tend to have fairly differing views on the time a client needs for recovery and also differ in the extent to which they take account of personal circumstances. Some take no account of this and others apply as a criterion that the client should not be exhausted after finishing work or that the client should be properly rested when he resumes work the next day [49,51].

### Social norm

In the case of insurance physicians the influence of social norms may emanate from the office and the environment. Within the office the norm for the 'strictness' of the assessments may differ [37]. Other factors are how much pressure is brought to bear in terms of production and promptness targets and how strictly these are checked [38,39].

Changing social norms can influence assessments [52]. The stricter the norm that states that society's interest should be guarded, the higher the probability that insurance physicians will find a sufficient job for the client and the higher the residual earning capacity. However, if the norm to which insurance physicians are subject states that society is responsible for its citizens if they become incapacitated for work, the probability that sufficient jobs are available will be lower [38]. In addition, Moore et al. [53] concluded that fear of claims can influence the relationship with the client and hence the decision as well. If the physician is afraid that the client will appeal, he will be less inclined to rule against the client's wishes [54]. Insurance physicians who are confronted by stricter medical professional norms are more inclined to decide that the client is no longer able to perform any work whatever [38].

### Self-efficacy

Self-efficacy (a person's belief about his ability and capacity to accomplish a task) moderates behaviour [24].

### Barriers

Work pressure, autonomy, emotional workload and emotional exhaustion are known to be related to health complaints and influence job participation [55-57]. Office culture can strongly influence job satisfaction and hence either hinder or stimulate the physicians in their work.

Factors connected with quality are also important. In the case of insurance physicians, managerial emphasis on quantity can act as a barrier [58]. Some physicians consider that the quality of the assessments suffers from work pressure and changes in the organisation. Lack of time means that duties are carried out with less care [49,50]. Choij [59] concludes that the attitude of professionals to the organisation is negative. They feel that they are not heard, recognised or appreciated. Management is seen as the major culprit because it exerts undue production pressure. Stricter requirements and an increase in the number of clients with more serious sicknesses reduces the physician's feelings of autonomy and increases stress through time pressure [60]. Guidelines and protocols can be an aid in enhancing quality [61]. However, physicians consider that they receive too little guidance in applying guidelines for disorders that are difficult to assess objectively [62] or in applying legislation, regulations and instruments [50]. 40% of the files are not in keeping (or completely in keeping) with the statutory requirements [63].

More difficult assessments may result in more variation between insurance physicians. The assessment is perceived as more difficult in relation to certain groups of clients. This applies to older clients [37,64], clients with mental problems [37,39], clients with impairments that are difficult to determine objectively [65,66] and clients with psychosomatic disorders [64]. Interviews with insurance physicians during the preparation of this questionnaire also showed that they regard assessment of the following categories as extra difficult: clients with a poor command of Dutch, clients who act aggressively or manipulatively, and cases where poor preliminary work has been done by the occupational health service.

### Knowledge

Deciding whether or not to request and use information of third parties may influence the assessment, and this is done to a very varied extent [62,67,68]. Davis et al. [69] found that diagnostic uncertainty among GPs was associated with higher rates of investigation and follow-up. Having more information increased the physician's self-confidence [43]. However, Kerstholt et al. [70] suggest that assessments of disability are largely based on the initial view formed after reading the file. The main pitfall is that the final view is based on general beliefs rather than on actual client information. The insurance physician's job experience, competencies and interests determine in part how difficult it is to 'translate' mental complaints into limitations and residual capacities [44]. Physicians need additional instruments, knowledge and experience in relation to disorders that are difficult to assess objectively [66].

### Intention

Studies by De Boer et al. [65] and Spanjer [71,72] show that the object, the physician's interpretation of his duties and the basic premises have an important bearing on the proposed assessment.

### Behaviour

We will distinguish two types of behaviour: 1) behaviour that reflects the process of assessment, such as the collection of information about the client and 2) behaviour directly connected with the assessment itself, such as the use of assessment instruments in order to evaluate the information.

Spanjer [18] found that process variables hardly affected assessment outcomes. Information on participation and activity limitations provided by the patient had only limited influence on inter-rater reliability by insurance physicians. However, there was a significant difference in scores on assessed work limitation items compared with medical history-taking alone. It follows that in disability assessment interviews physicians should ask for medical information as well as detailed information on participation and activity. Dedication (an aspect of 'work engagement' [73]) is a reflection of the intention to carry out tasks and is therefore placed under behaviour. This also applies to dealing with conflicts. A client may have a different view on the outcome of the assessment. This may result in a conflict during a disability evaluation. The above concepts are described in the model under 'Behaviour: process'.

Behaviour directly connected with the assessment itself was defined as the use of instruments to assess capacity for work and the importance the insurance physician attached to the client's opinion about his/her own functional capacities. In the Netherlands the insurance physician must determine the client's capacity for work. This is done by reference to an instrument known as FAL (Functional Ability List). On this list the physician enters the client's scores for limitations and abilities. These findings serve as the input for the labour expert in determining the extent to which the client is able to earn income and able to work. As an instrument the FAL comes within the statutory framework of disability assessments in the Netherlands. If the client is permanently and fully disabled, he is classified as such (in the Netherlands known as GBM). This is therefore a dichotomy measurement. The literature shows that statutory rules are implemented in different ways [74]. Insurance physicians interpret the guideline on permanent disability in a wide variety of ways [49,75]. The definition given in the guideline - namely an incapacity to function socially and personally - is considered inadequate. The criterion of permanent full disability is also used as a safety net in order to compensate for loss of income in difficult situations. The possibility of indicating that the client can work a limited number of hours (limitation of hours) is also applied in a wide variety of ways [45,49]. Likewise, the time required for recovery is assessed in a variety of ways [49]. The above concepts are described in the model under 'Behaviour: assessment'.

### Aim

The first aim of this study was to develop measurement instruments that can potentially affect disability assessment behaviour by insurance physicians. The description of a conceptual model includes the relevant (behavioural) variables. We expect that this model will contribute towards understanding and explaining variation in assessments of functional capacity by insurance physicians. A second aim was to discuss the descriptive results of the insurance physicians' scores on the different concepts. In accordance with these aims, the research questions were: 1) can we construct measurement instruments to measure assessment behaviour and its potential determinants? and 2) What are the characteristics of the assessment behaviour of Dutch insurance physicians and its potential determinants according to these instruments?

## Methods

### Study procedure

The research group of the organisations participating in this study - TNO Quality of Life, the EMGO Institute of the VU Medical Centre and the Employee Benefits Insurance Agency (UWV) - drafted the questionnaire for insurance physicians. At the start of 2008 UWV drew up a list of addresses of all insurance physicians working for the agency. In March 2008 UWV sent the questionnaire, together with a covering letter containing an invitation to participate in the research, to the home addresses of 750 insurance physicians. A reminder was sent two weeks later. Not all the physicians belonged to our target group, but it was not possible to make a selection in the mailing. In total we wrote to 750 insurance physicians. Our estimate is that the target group consisted of 450 insurance physicians. The criteria for inclusion were mentioned in the accompanying letter. We only included insurance physicians who were actively employed by UWV in May 2008 and who had also performed work disability assessments in 2007 or in preceding years. The participants sent the completed questionnaire to TNO (Netherlands Organisation for Applied Scientific Research). The response consisted of 231 questionnaires (estimated response approximately 51%). As this study was based on a survey under (insurance) physicians only, approval by a Medical Ethical Commission was not necessary under Dutch law.

### Questionnaire

In drawing up the questionnaire we used existing and newly developed concepts. These concepts were chosen on the basis of literature studies and four interviews with insurance physicians. In a pilot study two insurance physicians completed the questionnaire while thinking aloud in order to enable us to test whether the items were correctly understood. Finally, two other insurance physicians were timed while they completed the questionnaire. An English translation of the original Dutch questionnaire is accessible (additional file 1).

### Concept measurements questionnaire

#### Background variables

We measured gender, age, number of years' experience, training and specialisation. In the case of training and specialisation we included two items. First, we asked whether the insurance physician is registered as such (and is not still in training). Second, whether he practises or has practised in another area of medicine. In order to register differences between offices or regions we recorded the location of the insurance physician's office. We also asked how many hours they work each week, how many assessments they make each week, from which industry the majority of their clients come and the statutory background of the assessments of the majority of their clients, namely the Work and Income (Capacity for Work) Act (WIA), the Disability Insurance Act (WAO) or another statutory regime.

#### Attitude

Job satisfaction was measured by three items with five response categories ranging from (1) never to (5) always (I am satisfied with my work; my work suits me; I like my work) from Van Dijk et al. [76].

We included 11 items about the perceived justness of the social security system, the agency that administers the scheme (UWV) and the Permanent Full Disability Standard, FAL and the implementation of the Work and Income (Capacity for Work) Act (WIA). The items have five response categories, ranging from (1) I totally disagree to (5) I totally agree.

We included nine items on the attitude to quality in relation to the importance which insurance physicians attach to the development of skills, to refresher training, to guidance by management, to the development and use of protocols and guidelines and to updating the case file. The items have five response categories ranging from (1) I totally disagree to (5) I totally agree.

We included six items on the physician's attitude towards recovery time, the personal circumstances of the client and the physician's efforts to build a good relationship with the client. The items have five response categories ranging from (1) I totally disagree to (5) I totally agree or (1) never to (5) always.

#### Social norm

In the case of insurance physicians the influence of social norms may emanate from the office and the environment. We included four items on management attitudes to quantity as opposed to quality, to the use of protocols and guidelines and to production and outcomes. The items have five response categories ranging from (1) I totally disagree to (5) I totally agree.

In addition we included 13 items on the importance which insurance physicians attach to the exercise of their profession, to the opinion of the Employee Benefits Insurance Agency (UWV), the government authorities and professional organisations such as the Dutch Association for Insurance Medicine (NVVG) and the Dutch Association for Insurance Physicians at the UWV (UWVA), friends and family, colleagues in the office and elsewhere, public opinion, professional publications, quality assessment and the trade unions. The items have four response categories, ranging from (1) not important to (4) very important. The higher the score, the greater the importance attached to this opinion or view.

#### Self-efficacy

Self-efficacy was measured by the ten items formulated by Scholz et al. [77], adjusted to measure the insurance physician's belief about his ability and capacity to carry out work disability assessments. The items relate specifically to self-efficacy during the disability assessment interview. The items have four response categories, ranging from (1) completely incorrect to (4) completely correct.

#### Barriers

Work pressure was measured by means of a four-item scale drawn up by Smulders, Andries and Otten [78]. A sample item is 'Do you have to get through a lot of work?' A four-point answering scale was used ranging from (1) never to (4) always.

Emotional workload was measured using a three-item scale from the Copenhagen Psychological Questionnaire [79]. A sample item is: 'Does your work put you through emotionally difficult situations?'. Answers were scored on a four-point scale ranging from (1) never to (4) always.

Decision-making authority (4 items) was measured using a Dutch version of the Job Content Questionnaire, aimed at assessments [80,81]. A sample item is: 'Do you determine the order in which you carry out your tasks?' Answers were scored on a four-point scale ranging from (1) never to (4) always.

Emotional exhaustion was measured using the five-item emotional exhaustion scale of the Dutch version of the Maslach Emotional Exhaustion Inventory [82]. Answer categories varied from (1) never to (5) almost daily. The higher the score, the greater the exhaustion.

We included 12 items on cooperation, office atmosphere, consultation, being taken seriously by the management, and influence on workload. [76]. The higher the score the greater is the extent of the cooperation or co-determination. The items have five response categories ranging from (1) I totally disagree to (5) I totally agree.

We included 11 items on factors that could hinder or promote the quality of the assessment: legislation, reorganisations, support and guidance by staff physicians and management, reporting requirements, protocols/guidelines and standards, production requirements, refresher training and other measures to promote expertise, and mutual consultation. Each of the items has three response categories: adverse influence, no influence or beneficial influence.

We included 16 items concerning 'difficult clients'. We asked whether the following eight categories constitute an important proportion of the physician's clients and whether the physician considers the assessments of these categories to be extra difficult: clients with disorders that are difficult to determine objectively, clients with mental disorders, clients with a poor command of Dutch, clients who are aggressive, clients who are manipulative, clients who have problems at home or work, older clients and cases in which poor preparatory work has been done by the occupational health service.

#### Knowledge

In order to form a picture of the need for knowledge/information, the actual information received and the use of this information for the purposes of the assessment we choose to include 11 general items (i.e. not specifically relating to diagnosis) as to whether physicians had sufficient medical knowledge, medical information, information from the occupational physician (company doctor) regarding the attempt to return to work, the diagnosis and information from the parties. We also asked whether information from the reintegration report (drawn up by the occupational physician and sent with the WIA benefits application) was decisive and whether the physician received sufficient feedback from the claims manager about the outcome of his assessments. The items had four response categories, ranging from (1) never to (4) always.

#### Intention

For practical reasons we chose to measure only 'the object, the physician's interpretation of his duties and the basic premises' in relation to intention. A questionnaire in which intention is measured in respect of all behavioural items would be much too long. The 15 items had five response categories ranging from (1) not at all important to (5) very important. In the case of object/interpretation of duties we asked how important the following objects are in relation to the assessment: determination of physical capacities and cause of sickness, promotion of behaviour conducive to recovery, return to work, client's self-insight and reintegration. We also asked how important the following factors are in the assessment of claims: health complaints, impairments, limitations or handicaps of the client, an internally consistent and plausible account provided by the client, thorough questioning of the account given by the client of his daily activities, work capacity, chances in the labour market and information about the client's home situation.

#### Behaviour process

Engagement is a concept that refers to being fully immersed in an activity (absorption), being highly activated (vigour) and identifying with the work (dedication). We used the four items of the subscale of dedication on the engagement scale developed by Schaufeli et al. [73]. The items have five response categories, ranging from (1) never to (5) always. The higher the score, the greater is the work dedication.

Research by De Boer et al. [83] shows that although there are various interview models, they are not used as such. We did not therefore ask about the models, but included nine items on different core elements from the models, such as who determines what is discussed in the interview (physician or client), whether the physician asks questions in a fixed order, whether the physician asks questions about subjects raised by the client, and whether the physician asks for concrete examples of barriers and examines whether barriers result in limitations. The items had four response categories, ranging from (1) never to (4) always.

To measure conflict handling we used the Dutch Test for Conflict Handling [84], after modifying the items to confine them to the disability evaluation and conflicts with a client. This test measures to what extent five strategies are applied for handling conflicts, namely yielding, problem-solving, compromising, avoiding and forcing. We used three items for each strategy (total 15 items).

#### Behaviour assessment

As far as the 'permanent full disability' criterion is concerned we included five items on the extent to which the rules are followed and how physicians assessed a client who is completely unable to work but can still function in at least one social role. The items had four response categories, ranging from (1) never to (4) always.

As regards the FAL we included nine items in order to estimate to what extent physicians focus on a) limitations, impairments or complaints; b) what the client can do; c) difficult home circumstances; d) internal and external consistency; e) worsening health; and f) consultation with the labour expert. The items had five response categories, ranging from (1) I totally disagree to (5) I totally agree.

As regards behaviour in relation to the client, it is evident from the study by Nagtegaal [85] that the client's account of daily activities is a useful instrument in assessing the extent of his physical capacities. We included 10 items with four response categories, ranging from (1) never to (4) always. We asked how often the interview lasted as long as necessary, whether the client was treated with respect, whether the physician felt involved with the client, whether the physician took an independent position and did not allow himself to be affected by the client's interests and whether the physician took the time to question the client thoroughly about his account of his daily activities, to provide good reasons for his conclusion and to write a good report.

### Analyses

#### Response

In total we wrote to 750 insurance physicians. Our estimate is that the target group consisted of 450 insurance physicians. The response consisted of 231 questionnaires (estimated response approximately 51%). As we lacked the necessary data of the target population to do a full non-response analysis, we checked whether the group of participants (N = 231) was representative of the total population of insurance physicians working for UWV (N= approximately 900, including staff-members and physicians not performing disability assessments) in terms of age, gender, and working hours per week. The mean age and 95% confidence interval (CI) of the respondents was 50.8 years (95% CI [49.1;51.7]) and 41.1% were female. The respondents worked on average 32.5 hours per week (95% CI [31.5-33.4]). The total population's mean age was 49 years and 41.7% were female. Insurance physicians of the total population worked on average 32 hours per week. Although distribution measures of these population means could not be calculated, even if the (unknown) population confidence intervals were smaller than those of the respondent group, the respondent group in our study would not significantly differ from the population of insurance physicians in terms of age, gender, and working hours per week.

#### Imputation of missing values

With listwise deletion, only 122 cases of the 231 cases would be left. We therefore decided to impute for missing values. Because year of birth was not answered in 40 of the 231 cases, we imputed 38 of these missing cases by the predictions of an OLS regression equation for age (i.e. year of birth minus 2008). In the regression equation (listwise, enter procedure, n = 185) we used the other background variables as independent variables: sex (dummy), registered as insurance physician (dummy), (formerly) registered as curative specialist (dummy), working hours per week, number of assessments per week, type of statutory scheme applicable to most of the assessments (three dummies for WIA, WAO and the Invalidity Insurance (Young Disabled Persons Act (Wajong)) and sector (ten dummies for eleven sectors). The multiple correlation of the predicted age with the observed age was 0.696; the standardised residuals had a completely normal distribution. The SPSS 15.0 program [86] was used for this regression analysis. The remaining missing values for the background variables, the scale variables and the object scores of the HOMALS dimensions (see the next paragraph) were imputed using the 'expected maximisation' algorithm [87]. There were three variables with eleven to seventeen imputed cases, six variables with six to ten imputed cases and thirteen variables with two to five imputed cases. The remaining variables had no or only one imputed case. The interactive Lisrel program with Prelis 2.72 [88] was used for this imputation procedure.

#### Construction of scales and dimensions for the ASE concepts

The answers of the 231 insurance physicians were used to determine which concepts from the questionnaire were suitable for further analysis. The responses given by the insurance physicians were inspected. For some items it was necessary to recode the original items in fewer categories as some categories were empty or almost empty. Negatively formulated items were recoded positively.

Scales were formed for the following already validated scales: job satisfaction, self-efficacy, work pressure, emotional workload, decision-making authority, emotional exhaustion and engagement. Cronbach's alpha was computed for each of these scales. For the remaining items, factor analyses with principal components analysis and varimax or oblique rotation per block of items, were performed to extract factors for each theoretical concept. Oblique rotation was chosen only if there was a significant correlation between the extracted factors. Where this was not the case, we decided to use varimax rotation. Bartlett's test of sphericity was used to test whether the correlation matrix was an identity matrix. The sampling adequacy was inspected by means of the Kaiser-Meyer-Oikin measure (KMO) and found to be greater than 0.6. The number of extracted factors was decided on the basis of the scree test, the Eigenvalue and, most of all, the interpretability of the extracted factors. For each extracted factor, reliability analysis, including item analysis, was performed to construct additive scales from the items of the factors. An additive scale is constructed of numerical categories of items that can be meaningfully added. In the item analysis, the contribution of each item to the reliability of an additive scale can be estimated. If an item did not contribute to an additive scale, this item was deleted from this scale. When Cronbach's alpha was equal to or larger than 0.6, additive scales of the selected items were calculated. We nonetheless also decided to use additive scales in three cases where Cronbach's alpha was less than 0.6 (0.560, 0.566 and 0.594, respectively). These three scales were considered to be theoretically important. For each additive scale we also calculated the percentage of respondents who, on average, scored above the theoretical mean of the additive scale. This means that in case of an additive scale consisting of four Likert scale items ranging from 1 to 5, we report the percentage of respondents with a scale average above 3*4 = 12.0. In the remaining text when we refer to scales, we mean 'additive scales'.

For some blocks of items and for some individual items it was not possible to construct a scale for several reasons: the correlation matrix was not an identity matrix, and/or the sampling adequacy was not good, or Cronbach's alpha was too small. We grouped these 'lost' items on a theoretical basis, recoded them, if necessary, into two or three categories and used HOMALS (homogeneity analysis by means of alternating least squares) to analyse the dimensions behind these grouped items [89]. The number of dimensions was decided on basis of the sum of the Eigenvalues of the dimensions. We estimated for each dimension the discrimination measures of the items, the category quantifications of categories of items, and the object scores of the cases. We used the discrimination measures and the category quantifications to interpret both poles (negative and positive) of the dimensions. The object scores of the dimensions that were meaningful and gave additional information were selected as variables. Because object scores of multiple Homals dimensions are constructed with non-linear transformations [90], they are not scales, and reliability analysis cannot be performed. Therefore, we call these variables 'dimensions', contrary to the variables which we constructed as additive scales, which we call 'scales'. We used the SPSS 15.0 program [86] for the factor analyses, reliability analyses and the HOMALS analyses.

## Results

**Figure 2:**
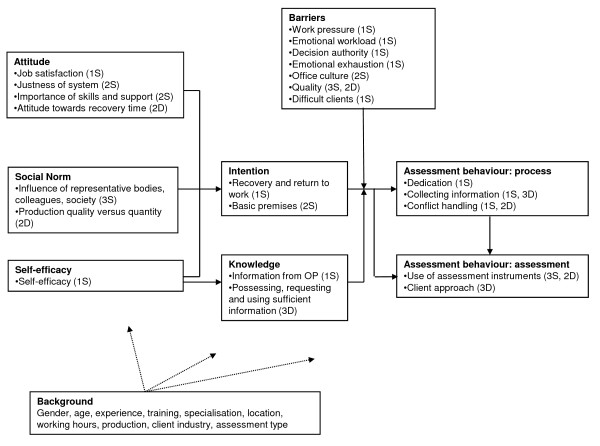
**The ASE model with a summary of scales and dimensions**. S = Scale; D = Dimension, the number refers to the number of constructed scales and dimensions.

Descriptives of all measured scales are presented in table 1 and those of the measured object scores resulting from the HOMALS analyses in table 2, including the final number of items. When not all original items are included in the scales and dimensions, we report it in this section. A summary of scales and dimensions for each ASE concept is presented in figure 2.

**Table 1:** Description of scales (n = 231)

ASE	Scale	# items	% yes/high^1^	Theor. max^2^	Median	Mean	sd	Cronbach's alpha
Attitude								
	Job satisfaction	3	78	15	12	11.41	2.51	0.875
	Positive attitude towards WIA	5	53	25	16	15.98	3.63	0.797
	Social security system just	5	70	25	17	17.43	3.25	0.636
	Quality: development of skills important	5	99	25	22	22.11	2.27	0.648
	Quality: support by management important	3	68	15	11	10.46	2.32	0.643
Social Norm								
	Opinion of UWV and employee representative bodies important	6	43	24	15	15.01	2.75	0.697
	Colleagues' opinion important	5	66	20	14	13.40	2.36	0.679
	Society's opinion important	3	10	12	6	5.77	1.53	0.560
Self-efficacy								
	Self-efficacy	10		40	32	32.81	4.21	0.908
Barriers								
	Work pressure	4	44	16	10	10.18	2.06	0.771
	Emotional workload	3	20	12	6	6.38	1.42	0.702
	Decision making authority	4	61	16	11	10.96	2.65	0.724
	Emotional exhaustion	5	12	25	10	10.79	3.97	0.892
	Office culture: good cooperation	8	83	40	30	29.62	5.65	0.900
	Office culture: sufficient co-determination	4	20	20	10	9.78	3.25	0.814
	Quality: influence of refresher training and consultation beneficial	2	97	6	6	5.90	0.38	0.665
	Quality: influence of staff physician beneficial	2	80	6	6	5.38	0.89	0.675
	Quality : influence of manager beneficial	2	27	6	4	4.03	1.05	0.647
	Many difficult clients/cases	16	73^3^		16	15.62	2.82	0.675
Knowledge								
	Sufficient information from the occupational physician	3	44	12	7	7.20	1.33	0.769
Intention								
	Stimulate recovery and return to work	4	94	20	17	16.71	2.77	0.852
	Basic premises: residual capacity	6	99	30	28	27.03	2.75	0.809
	Basic premises: client's account and home circumstances	4	97	20	18	17.37	2.17	0.727
Behaviour Process								
	Dedication	4	73	20	14	13.94	2.74	0.874
	Technical interview: describe object and procedure	2	84	8	7	6.71	1.15	0.594
	Conflict handling: seek compromise	7	5	35	15	15.57	3.46	0.733
Behaviour Assessment								
	Comply with permanent full disability rules	2	72	8	6	6.09	1.23	0.734
	FAL: take account of client	5	31	25	14	14.16	2.47	0.566
	FAL: consult with labour expert when not necessary	2	63	10	8	7.09	2.00	0.761

^1^yes/high = % of respondents whose score is above the theoretical scale average, e..g. the % of respondents who on average '(totally) agree', think of something as '(very) important', or score 'often/always'.

^2 ^The theoretical maximum value of the scale.

^3 ^The percentage of insurance physicians who classify the majority of their clients/cases as difficult.

**Table 2:** Description of Homals object scores for dimensions (n = 231)

	# items	Min	Max	Median	Mean*	Sd*	Eigen value
Attitude							
Recovery time: client still has some energy left after work	6	-2.50	1.79	-0.0784	0.00	1.00	0.254
Recovery time: good relationship with client	6	-3.36	2.07	0.0931	0.00	1.01	0.188
Social norm							
Managing by reference to quality rather than quantity	4	-1.44	1.70	-0.0953	0.00	1.00	0.489
Managing less by reference to production targets and outcomes	4	-1.16	4.03	-0.1753	0.01	1.00	0.371
Barriers							
Quality: influence of legislation and reorganisations not adverse	4	-1.75	2.13	-0.0833	0.00	1.02	0.352
Quality: influence of guidelines not adverse and production target not beneficial	4	-1.25	2.90	-0.6381	0.01	1.02	0.269
Knowledge							
Possessing, requesting and using insufficient information	8	-2.65	2.20	0.0174	-0.01	1.02	0.231
Insufficient medical information and knowledge	8	-2.77	1.80	0.0751	0.00	1.01	0.173
Sufficient knowledge, reintegration report less often supplements medical information	8	-2.67	2.77	-0.0985	0.00	1.01	0.154
Behaviour Process							
Interview management: client decisive	6	-1.66	2.46	-0.2199	0.00	1.00	0.263
Interview: limitations not checked	6	-1.32	3.09	-0.4262	0.00	1.00	0.214
Interview: respond to client	6	-2.32	2.61	-0.0496	0.00	1.00	0.170
Conflict handling: engage in confrontation	8	-1.91	2.63	0.0086	0.00	1.00	0.235
Conflict handling: play down differences	8	-1.92	2.87	-0.1829	0.00	1.00	0.209
Behaviour Assessment							
FAL and recovery time: strict/formalistic approach	6	-9.13	1.82	0.0851	-0.07	1.27	0.292
FAL and recovery time: focus on impairments	6	-8.94	2.27	0.0507	-0.28	1.65	0.288
Client approach: involved with and time for	8	-2.24	2.25	-0.0536	0.00	1.00	0.263
Client approach: time for account of daily activities and reporting	8	-2.68	1.58	0.0614	0.00	1.00	0.178
Client approach: too little time, but involved with	8	-7.66	2.74	-0.2466	-0.07	1.23	0.168

* Because of imputation, object scores can deviate from mean = 0 and sd = 1.

### New scales and dimensions

#### Attitude

We developed two scales on the theme of justness (10 of the 11 original items): The higher the score on the scale 'Positive attitude towards the WIA', the more positive the opinion about the justness of the WIA. The higher the score on the scale 'Social security system is just', the more positive the opinion about the agency that administers the scheme (UWV), the Permanent Full Disability Standard and FAL.

We developed two attitude scales for quality (eight of the nine original items). The higher the score on the scale 'Quality: development of skills important', the greater the importance attached by the physician to the promotion of expertise, consultation with colleagues, working in accordance with protocols and properly updating the case file. The higher the score on the scale 'Quality: support by management important', the greater the importance attached by the physician to support and management by the immediate superior and support by the staff physician.

We developed the two attitude dimensions on recovery time (six items). The higher the score on the 'Recovery time: client still has some energy left after work' dimension, the more the insurance physician agrees the client should not be completely exhausted after work. The higher the score on the 'Recovery time: good relationship with client' dimension, the more the insurance physician tries to establish good relations with the client and takes account of personal circumstances.

#### Social norm

We developed three scales (12 of the 13 original items) and two dimensions (four items). The following three scales are about the influence which the opinions and views of certain persons/authorities have on the performance of the profession. The higher the score on the scale, the greater the importance attached to this opinion or view. The 'Opinion of UWV and employee representative bodies important' concerns the influence of the UWV (supervisor, staff physician, objection and appeal), and employee representative bodies such as the Dutch Association for Insurance Medicine (NVVG), the Dutch Association for Insurance Physicians at the UWV (UWVA) and the trade union. The 'Colleagues opinions important' scale concerns the influence of fellow insurance physicians, both in the office and elsewhere. The 'Society's opinion important' scale concerns the influence of family, friends, TV and government.

The higher the score on the dimension 'Managing by reference to quality rather than quantity', the more importance is attached in relative terms to quality-based management and the less importance to quantity-based management. The higher the score on the dimension 'Managing less by reference to production targets and outcomes', the less importance is attached to management based on production targets and outcomes.

#### Barriers

We developed four scales and two dimensions for barriers. Of the 11 original items about quality of the assessment, six were used in three scales of two items each and four were used in two dimensions. The higher the score on the 'Quality: influence of staff physician is beneficial' scale, the more the assessment benefits in terms of quality from support and professional guidance provided by the staff physician. The higher the score on the 'Quality: influence of refresher training and consultation beneficial' scale, the greater the beneficial effect of refresher training, mutual consultation and measures to promote expertise support. Finally, the higher the score on the 'Quality: influence of manager beneficial' scale, the greater the beneficial effect of the support and professional guidance provided by the manager.

We constructed one scale named 'Many difficult clients', based on 16 items. The higher the score, the more the insurance physician is confronted with clients whom he experiences as difficult.

The higher the score on the 'Quality: influence of legislation and reorganisations not adverse' dimension, the lower the adverse impact of legislation and UWV reorganisations on quality. The higher the score on the 'Quality: influence of guidelines not adverse and production requirement not beneficial' dimension, the lower the adverse impact of the guidelines and the lower the beneficial impact of the production requirement on quality.

#### Knowledge

We developed one scale (three items) and three dimensions (eight items). The higher the score on the 'Sufficient information from the occupational physician' scale, the more sufficient the available information from the OP. The higher the score on the ' Possessing, requesting and using insufficient information' dimension, the more the physician considers that he does not always have sufficient medical information available, does not always request information from third parties and does not always take this into account in making the assessment. The higher the score on the 'Insufficient medical information and knowledge' dimension, the more the physician considers that he does not always have sufficient medical information and medical knowledge to make the assessments. The higher the score on the 'Sufficient knowledge, reintegration report less frequently supplements medical information' dimension, the more sufficient is medical information and the less frequently the reintegration report supplements the medical information.

#### Intention

We developed three intention scales (14 of the original 15 items). The higher the score on these scales, the more importance is attached to the subjects. The 'Stimulate recovery and return to work' scale concerns the importance to promote recovery behaviour, return to work, self-insight and reintegration in the assessment. The 'Basic premises: residual capacity' scale concerns the importance of residual capacity, sickness, impairments, limitations and handicaps in the assessment. Finally, the 'Basic premises: client's account and home circumstances' scale concerns the importance of a consistent account of daily activities, thorough questioning about this account and information about the client's home circumstances in the assessment.

#### Behaviour process

We developed one scale and three dimensions concerning the collection of information about functional capacities (eight of the nine original items). The higher the score on the 'Technical interview: describe object and procedure' scale, the greater the emphasis which the physician puts at the beginning of the interview on the purpose of the interview and the procedure to be followed during it. The higher the score on the 'Interview management: client decisive' dimension, the more the client determines the order of events rather than the insurance physician. The higher the score on the 'Interview: limitations not checked' dimension, the less frequently the insurance physician checks what limitations the client faces. The higher the score on the 'Interview: respond to client' dimension, the more frequently the insurance physician responds to subjects raised by the client.

The Dutch Test for Conflict Handling [84] measures to what extent five strategies are applied for handling conflicts, namely yielding, problem-solving, compromising, avoiding and forcing. It is noteworthy that insurance physicians evidently did not use all five of these strategies during the disability evaluation. We were able to measure one scale (based on seven items) and two dimensions (calculated from the remaining eight items). The higher the score on the 'Conflict handling: seek compromise' scale, the more often the physician searches for a compromise with a client in the event of a difference of opinion. The higher the score on the 'Conflict handling: engage in confrontation' dimension, the more likely the physician is to engage in confrontation with the client in the event of a difference of opinion. The higher the score on the 'Conflict handling: play down differences' dimension, the more often the physician will try to circumvent differences of opinion and play down their importance.

#### Behaviour assessment

We developed three scales and five dimensions (23 of the 24 original items). The higher the score on the 'Comply with permanent full disability rules' scale, the more strictly the insurance physician complies with the permanent full disability rules. The higher the score on the 'FAL: take account of the client' scale, the more often the physician focuses on the complaints raised by the client, what the client can really do, the client's difficult home circumstances and limitations experienced by the client. The higher the score on the 'FAL: consult with labour expert when not necessary' scale, the more often the insurance physician will consult with the labour expert in circumstances where the client is unable to work or does not belong in the benefits category.

The higher the score on the 'FAL and recovery time: strict/formalistic approach' scale, the more the insurance physician takes a formalistic and strict approach to drawing up the FAL and takes no account of the client and his recovery time. The higher the score on the 'FAL and recovery time: focus on impairments' scale, the greater the attention which the insurance physician pays when drawing up the FAL to limitations caused by impairments, particularly in the light of consistency, and takes no account of a possible deterioration in the client's health.

The higher the score on the 'Client approach: involved with and time for' dimension, the more often the insurance physician takes time for and is involved with the client. The higher the score on the 'Client approach: time for account of daily activities and reporting' dimension, the more often the insurance physician takes time to thoroughly question the client about his daily activities and to report on this. The higher the score on the 'Client approach: too little time but involved with' dimension, the more likely it is that the insurance physician has too little time to draw up a proper report and to question the client about his daily activities, but feels involved with the client.

### Descriptive results

Descriptive results for background variables are summarised in table 3. 58.9% of the insurance physicians in this survey were men. On average the respondents were aged 50.8 years and had 16.2 years of experience. 85.7% were registered and almost two third worked 33 or more hours per week and carried out on average 9.1 disability assessments per week. The patients came from all industries. It is noteworthy that 53.7% of the insurance physicians reported that a substantial proportion of their clients were temporary workers.

**Table 3:** Background variables

	%	mean	sd
Gender (%man)	58.9		
Age		50.8	7.0
Registered as insurance physician	85.7		
Extra medical speciality	15.2		
Working hours (week) % up to 24 hrs	16.0		
% 25-32 hors	23.8		
% 33 hrs or more	60.2		
N assessments (week)		9.1	4.0
Years of experience		16.2	7.7
Assessments mainly under WIA	37.7		
Assessments mainly under WAO	26.4		
Assessments mainly under Wajong	13.0		
Clients mainly from the agriculture, fishing and food industries	13.0		
Clients mainly from the construction and timber industries	19.5		
Clients mainly from manufacturing industry	39.4		
Clients mainly from the retail and wholesale sectors	41.6		
Clients mainly from the transport sector	24.2		
Clients mainly from the financial services sector	26.8		
Clients mainly from the temporary work sector	53.7		
Clients mainly from the health sector	35.1		
Clients mainly from the education sector	22.1		
Clients mainly from the rest of the public sector	13.0		
Clients mainly from the professions and other sectors	33.8		

As regards attitude, table 3 shows that 78% of the insurance physicians were motivated by the job, 70% considered that the social security system was just and only 53% had a positive opinion about the WIA. The results for 'social norm' show that insurance physicians are mostly influenced by colleagues (66%) and by their employer (UWV, 43%) and much less by society/the public (10%). The results on barriers show that 44% of the insurance physicians experienced substantial work pressure and 20% substantial emotional workload, 12% were emotionally exhausted and 73% reported that they viewed the majority of their clients/cases as 'difficult'. The influence of refresher training and the staff physician is viewed as conducive to quality, whereas only 27% of the insurance physicians consider that the manager promotes quality. As far as knowledge is concerned, less than half of the physicians consider that they receive sufficient information from the occupational physician (company doctor). The scores for intentions show that most insurance physicians intend to carry out the profession in the manner expected of them as professionals. As regards behavioural process, we see that three quarters of the physicians are dedicated and inform the client of the object of the interview and the procedure and that only 5% indicate that they seek a compromise in the event of a difference of opinion with the client. As regards behavioural assessment, we see that 72% of the insurance physicians follow the rules, 31% consider that they have taken account of the client and 63% frequently consult with the labour expert in circumstances where this is not mandatory, namely in situations of 'medical incapacity for work' or 'capacity for own work'.

## Discussion

### Discussion of the methods

In this article we have presented the development of instruments for measuring and explaining variations in the behaviour of insurance physicians in relation to assessments of functional capacities. Data from 231 questionnaires were analysed and used as a basis for filling the ASE model with 29 scales and 19 dimensions. We identified scales and dimensions that represent Attitude, Social norm, Self-efficacy, Barriers, Knowledge and Intention. We slightly modified the underlying ASE model by dividing Behaviour into two blocks, the first reflecting the process and the second reflecting assessment-related behaviour. The value of the instruments proposed in this article lies in their specificity for insurance physicians and their sound psychometric characteristics. The extensive literature study, in combination with the interviews safeguarded the internal validity.

While our instruments and the underlying concepts show considerable similarities to the study of the communication of insurance physicians with their clients conducted by Van Rijssen et al. [91], the operationalisation of the underlying concepts was specifically designed to meet the objective of the present study. Our analysis model is an extension of the model designed by Croon and Langius [29] in their study of the process of sickness certification assessment by social insurance physicians. They took the theory of planned behaviour as a starting point. The concept of barriers and stimuli experienced by physicians, their own effectiveness and the availability of sufficient knowledge (concepts which are recognised in the ASE model) are also included in their model. Our analysis model divides Croon and Langius' concept of the 'influence of the environment' into the concept of the social norm (which influences the intention) and barriers (which have an intermediary effect between intention and behaviour). It could be argued that the conceptual model of the theory of planned behaviour is problematic in that its concepts are not specific enough [92]. We have countered this argument in our proposed model by focusing the concepts specifically on the subject of work disablement assessment.

One particular strength of this study is the extent of the good response to the survey by the insurance physicians, which was considerably higher than we had expected. A weakness of the study is its cross-sectional design, which does not allow for analysis of causal relationships between attitude, social influence, intention and behaviour. Another weakness may be the fact our explanation of measured scales and dimensions in relation to the ASE concepts is only based on theoretical grounds. It is therefore possible that certain scales and dimensions may not fit in with the ASE concept. Furthermore, the study does not investigate the structural relationships between the measured constructs. Further study is therefore needed in order to demonstrate whether the ASE model is the best model to explain insurance physician's behaviour.

### Discussion of content

The descriptive results may give rise to some concern. We see a professional group that is highly motivated about the job and positive about the Dutch social security system. However, only half of them have a positive opinion about the Work and Income (Capacity for Work) Act (WIA). The views of the insurance physicians about the social security system and legislation are, in principle, separate from the manner in which they carry out their professional duties and endeavour to achieve a high quality. Furthermore, insurance physicians experience serious barriers, the most frequent of which is work pressure. Work pressure, emotional workload and emotional exhaustion are positively correlated. Finally, 73% of the insurance physicians describe a majority of their clients/cases as 'difficult'. In order to determine if these scores were relatively high, we compared our outcomes with the same scales of a large survey among employees (NEA 2008). This comparison reveals that the insurance physicians in this study do not differ significantly from the NEA group 'physicians, dentists and veterinary surgeons (N = 240)' in terms of work pressure, emotional workload and emotional exhaustion. Insurance physicians were found to have higher levels of autonomy than other 'physicians, dentists and veterinary surgeons'. The negative aspects do not therefore differ from those of a comparable group of professionals.

The answers to the questionnaire also indicate that insurance physicians are primarily bound, as regards their professional conduct, by the norms and views of insurance physicians as a professional group. In this way, frameworks are set for the discretionary power of the insurance physicians which is necessary in order to do justice in special cases. A fellow insurance physician must be able to come to the same assessment (reproducibility).

The management of UWV (Dutch Benefits Insurance Agency), which focuses above all on the work processes and production, is often seen as setting norms, but is not regarded as supporting the quality of the work. This is not a unique finding, but an illustration of the problem of managing professionals in general [93]. In his international comparative study into work disability assessments De Boer [94] also concludes that the professional definition of quality of evaluation of work disability is 'performance according to professional standards'. He emphasises that in the Netherlands the requirement of a fair trial is also a central part of the quality of claim assessment. We see this reflected in our results. The results for social norms show that the insurance physicians attach most importance to the views of their fellow professionals and thereafter to those of their employer (UWV). They attach the least importance to the views of society. Many insurance physicians believe that the quality of their assessments is positively influenced by good cooperation with colleagues, refresher training and consultation, as well as guidance by staff physicians. Many insurance physicians score highly in terms of following rules during the assessment, so that a fair process is possible, while only few insurance physicians indicate that their work style is to look for compromises in the event of a difference of opinion with the client. This would detract from their independent professional status and the requirements of a fair trial. Nonetheless, this does not prevent one third of the insurance physicians from indicating that they take account of the client's specific circumstances when drawing up the functional capacity assessment.

## Conclusions

The scales and dimensions developed appear to be valid and offer a promising basis for future research. The results suggest that the underlying ASE model, in modified form, is suitable for describing the assessment behaviour of insurance physicians and the determinants of this behaviour. The next step in this line of research should be to validate the model using structural equation modelling. Finally, the predictive value should be tested in relation to work disability assessment outcomes, i.e. grant or reject the claim.

## Competing interests

All authors declare that there are no financial or other relationships that might lead to a conflict of interest.

## Authors' contributions

RS participated in the design of the study and its coordination, developed the questionnaire, performed the statistical analysis and drafted the manuscript. AS participated in the design of the study, participated in developing the questionnaire, performed the statistical analysis and participated in drafting the manuscript. HM participated in the design of the study and its coordination, developed the questionnaire and participated in drafting the manuscript. JRA participated in the design of the study and participated in developing the questionnaire. HK participated in the design of the study and its coordination and provided the logistics for the questionnaire. JB participated in the design of the study and its coordination, participated in developing the questionnaire and drafting the manuscript. All authors read and approved the final manuscript.

## Pre-publication history

The pre-publication history for this paper can be accessed here:

http://www.biomedcentral.com/1471-2458/11/1/prepub

## Supplementary Material

Additional file  1 **Questionnaire insurance physicians**. English translation of the original Dutch questionnaire for insurance physicians. Click here for file
