# Supplementary material for: The development of instruments to measure the work disability assessment behaviour of insurance physicians
Source: BMC Public Health. 2011 Jan 3;11:1. doi: 10.1186/1471-2458-11-1 (PMC3086528; doi:10.1186/1471-2458-11-1)
Supplement: Additional file 1 — Questionnaire insurance physicians. English translation of the original Dutch questionnaire for insurance physicians. [file 1471-2458-11-1-S1.PDF]

QUESTIONNAIRE

Variation in assessment of functional abilities by insurance physicians

February 2008 (translated from Dutch)

This questionnaire has been drafted with reference to the Dutch disability insurance system. In the Netherlands, if you are partially or fully incapable of working after two years of illness, you may be eligible to receive a benefit under the Work and Income (Capacity for Work) Act (WIA). Disability assessments are carried out by insurance physicians. Most insurance physicians are employed by the UWV (*Uitvoeringsinstituut WerknemersVerzekeringen* = Institute for Employee Benefit Schemes).
The WIA succeeded the Disability Insurance Act (WAO) in January 2006. The WAO was not repealed by the WIA, but now applies only to those who were already receiving a WAO benefit on 1 January 2006. The Adapted Re-assessment Act (HERBO) was introduced in August 2004 for the reassessment of WAO benefits clients, i.e. the claimants (<50 years), on the basis of new, stricter criteria that put the emphasis on the client's residual functional capacities. These stricter assessment rules under HERBO also apply to the WIA. Young disabled people may be eligible to receive a benefit under the Invalidity Insurance (Young Disabled Persons) Act (Wajong). The WAO and WIA differ in the time of assessment. The WAO provides for assessments after one year of illness, whereas the WIA provides for assessments after two years of illness.

Introductory notes

- Please fill in this questionnaire yourself; do not confer with others.
- Do not hesitate too long if you are not sure of the right answer. Just fill in the first answer that comes into your head.
- Only give one answer to each question, unless indicated otherwise. Tick the box that is most applicable to your situation.
- Remember, there are no right or wrong answers. What we are interested in is your opinion.
- Please do not skip any questions.

Please return the completed questionnaire by 14 March 2008 to: Romy Steenbeek, TNO Quality of Life, Reply No. 518, 2130 WB Hoofddorp, The Netherlands.
You can use the enclosed reply envelope for this purpose.

Thank you for your cooperation!

**General questions**	
Sex	❑ Male<br>❑ Female
Year of birth	……
Specialism and medical background<br>(you may tick more than one box)	❑ Physician<br>❑ Registered insurance physician<br>❑ Trainee insurance physician<br>❑ G.P.<br>❑ Occupational physician<br>❑ Medical specialist<br>❑ Forensic physician<br>❑ Other (please specify) ………………….
Position (you may tick more than one box)	❑ Insurance physician<br>❑ Senior (regional) insurance physician<br>❑ Insurance physician charged with dealing with appeals
How many hours a week do you work as an insurance physician?	❑ Less than 12<br>❑ 12-24<br>❑ 25-32<br>❑ More than 32
How many years have you been working as an insurance physician?	……years
How many disability assessments or reassessments do you perform per week, on average?	……
You see most of your clients in the framework of:	❑ A WIA assessment<br>❑ A WAO reassessment<br>❑ A WAJONG reassessment<br>❑ A WAJONG assessment<br>❑ Other (please specify)……………….
Most of your clients come from the following sectors (you may tick more than one box):	❑ Agriculture, fisheries, food industry<br>❑ Construction and timber<br>❑ Industry<br>❑ Retail and wholesale<br>❑ Transport<br>❑ (Financial) services<br>❑ Temp agencies<br>❑ Healthcare<br>❑ Government (education)<br>❑ Government (other)<br>❑ Other trades and professions
Which UWV office do you work for?	❑ Alkmaar<br>❑ Almere<br>❑ Amsterdam<br>❑ Apeldoorn<br>❑ Arnhem<br>❑ Assen<br>❑ Breda<br>❑ The Hague<br>❑ Doetinchem<br>❑ Den Bosch<br>❑ Dordrecht<br>❑ Eindhoven<br>❑ Emmen<br>❑ Goes<br>❑ Groningen<br>❑ Haarlem<br>❑ Heerlen<br>❑ Helmond<br>❑ Hengelo<br>❑ Leeuwarden<br>❑ Leiden<br>❑ Nijmegen<br>❑ Rotterdam<br>❑ Tilburg<br>❑ Utrecht<br>❑ Venlo<br>❑ Zwolle<br>❑ Other (please specify)<br>…………………………………………..

**Objectives and task-setting**					
How important are the following objectives in your assessment?	Very unimportant	Rather unimportant	Neutral	Rather important	Very important
Determining client’s (residual) work capacity.	❑	❑	❑	❑	❑
Determining cause of illness.	❑	❑	❑	❑	❑
Assisting recovery.	❑	❑	❑	❑	❑
Assisting return to work.	❑	❑	❑	❑	❑
Promoting client’s insight into own situation.	❑	❑	❑	❑	❑
Promoting reintegration.	❑	❑	❑	❑	❑
Other (please specify) ……….	❑	❑	❑	❑	❑

**Criteria**					
How important are the following criteria for your claim assessment?	Very unimportant	Rather unimportant	Neutral	Rather important	Very important
Client’s medical complaints.	❑	❑	❑	❑	❑
Client’s disorders.	❑	❑	❑	❑	❑
Limits on client’s work performance.	❑	❑	❑	❑	❑
Client’s disabilities.	❑	❑	❑	❑	❑
An internally consistent and plausible story from the client.	❑	❑	❑	❑	❑
Getting a good picture of client’s day-to-day functioning.	❑	❑	❑	❑	❑
Client’s ability to work.	❑	❑	❑	❑	❑
Information about client’s work prospects (prospects of employment).	❑	❑	❑	❑	❑
Information about the client’s home situation.	❑	❑	❑	❑	❑

**Social security and UWV**					
Please indicate the extent to which you agree with the following statements:	Completely disagree	Disagree to certain extent	Neither agree nor disagree	Agree to certain extent	Completel y agree
It’s a good thing for people to help one another through the social security system.	❑	❑	❑	❑	❑
The reassessment procedure laid down in the aSB is (or was) fair.	❑	❑	❑	❑	❑
The WIA is fair.	❑	❑	❑	❑	❑
The WAO, prior to aSB, was fair.	❑	❑	❑	❑	❑
The UWV treats its clients fairly.	❑	❑	❑	❑	❑
The WIA (compared with the WAO):					
Promotes reintegration better.	❑	❑	❑	❑	❑
Is fairer.	❑	❑	❑	❑	❑
Leads to a decrease in medicalisation.	❑	❑	❑	❑	❑
Erodes the social security system.	❑	❑	❑	❑	❑

**Views on GBM classification** (GBM = *geen benutbare mogelijkheden* = permanently and fully disabled)					
If a client is classified as GBM (permanently and fully disabled**),** because of the inability to function properly on the personal as well as the social level, possibly as the result of a previous diagnosis of EPS (*ernstige psychische stoornissen* = severe mental disorders), it is required that he or she does not function in the following three social roles: self-care, family, and social contacts outside the family including work.					
	Completely disagree	Disagree to certain extent	Neither agree nor disagree	Agree to certain extent	Completel y agree
I find this decision fair	❑	❑	❑	❑	❑
		Never	Sometimes	Often	Always
How often does it occur, in your opinion, that the client is completely unable to work but can function adequately in at least one social role?		❑	❑	❑	❑
What would you do in such a case?					
Follow the rules		❑	❑	❑	❑
Classify the client as GBM (unable to function independently**)** anyway		❑	❑	❑	❑
Fill in an FML (functional capabilities list) with such strong limitations (e.g. in terms of hours) that it is very unlikely that suitable employment opportunities will be presented		❑	❑	❑	❑
Make my views clear in my report		❑	❑	❑	❑

**Views on FML** (*functionele mogelijkheden lijst* = functional capabilities list)					
	Completely disagree	Disagree to certain extent	Neither agree nor disagree	Agree to certain extent	Completely agree
The FML is a good instrument for describing the client's capabilities and limitations.	❑	❑	❑	❑	❑
When filling in the FML:	Never	Rarely	Sometimes	Often	Always
I concentrate on the limitations that are directly linked with the presenting complaints.	❑	❑	❑	❑	❑
I concentrate on the limitations that are directly linked with the disorders that have been diagnosed and/or observed.	❑	❑	❑	❑	❑
I concentrate on what I believe to be the client's real capabilities, independent of the complaints or disorders.	❑	❑	❑	❑	❑
I concentrate on the limitations that are in line with the health complaints	❑	❑	❑	❑	❑
I take pressures occurring in the home situation into account.	❑	❑	❑	❑	❑
I take future deterioration of the client's state of health into account.	❑	❑	❑	❑	❑
I take the client's perception of his or her limitations into account if I am unsure how to fill in the FML.	❑	❑	❑	❑	❑
I consult the occupational health officer if I think the client is unable to work.	❑	❑	❑	❑	❑
I consult the occupational health practitioner if I think the client does not qualify for a disability benefit.	❑	❑	❑	❑	❑

.

**Recovery time**					
The following questions are about the client's recovery time, by which we mean here the time the client needs to recover after work so as to keep demands and capacity in balance					
	Never	Rarely	Sometimes	Often	Always
How often do you take the recovery time required into account when determining the client's capabilities?	❑	❑	❑	❑	❑
Please indicate the extent to which you agree with the following statements about the recovery time.	Completely disagree	Disagree to certain extent	Neither agree nor disagree	Agree to certain extent	Completely agree
The client needs to have some energy in reserve at the end of the working day, for example in order to cope with family tasks and other social activities.	❑	❑	❑	❑	❑
The client should feel fresh at the start of each new working day.	❑	❑	❑	❑	❑
The client's personal circumstances (for example living alone, being the head of a single-parent family, having to care for young children or others) have an effect on the recovery time.	❑	❑	❑	❑	❑
How do you take the need for recovery time into account in the FML?	Never	Rarely	Sometimes	Often	Always
In category VI (hours of work)	❑	❑	❑	❑	❑
Under another heading	❑	❑	❑	❑	❑

**Groups where assessment can be difficult**		
Please indicate whether the groups given below represent a significant part of your case load (you see such clients at least once a week if you have a full-time position) and/or present particular difficulty in the claim assessment.	Makes up a significant part of my case load	Can be particularly difficult
Clients with a complaint that is difficult to objectify (e.g. chronic fatigue syndrome or ME, fibromyalgia, pelvic instability or whiplash injury).	❑ No ❑ Yes	❑ No ❑ Yes
Clients with a mental health condition such as stress or burn-out.	❑ No ❑ Yes	❑ No ❑ Yes
Immigrants or other clients with a poor command of Dutch.	❑ No ❑ Yes	❑ No ❑ Yes
Aggressive clients.	❑ No ❑ Yes	❑ No ❑ Yes
Manipulative clients.	❑ No ❑ Yes	❑ No ❑ Yes
Clients with problems at home or at work.	❑ No ❑ Yes	❑ No ❑ Yes
Clients that have not been properly processed by the Health & Safety service.	❑ No ❑ Yes	❑ No ❑ Yes
Older clients.	❑ No ❑ Yes	❑ No ❑ Yes
Others (please specify) ………………….	❑ No ❑ Yes	❑ No ❑ Yes

**Quality**			
Please indicate which of the following factors have a positive or negative influence on the quality of your claim assessments:	Hinders proper assessment	No influence	Promotes good assessment
Legislation.	❑	❑	❑
Reorganization at UWV.	❑	❑	❑
Support from senior insurance physician.	❑	❑	❑
Professional guidance by senior insurance physician.	❑	❑	❑
Support from direct superior.	❑	❑	❑
Guidance by direct superior.	❑	❑	❑
Minimal requirements on reporting.	❑	❑	❑
Protocols, guidelines and standards.	❑	❑	❑
Production requirements.	❑	❑	❑
Permanent education and training.	❑	❑	❑
Consultation with colleagues	❑	❑	❑

Please indicate the extent to which you agree with the following statements:	Completely disagree	Disagree to certain extent	Neither agree nor disagree	Agree to certain extent	Completely agree
I regard permanent education and training as important for keeping my skills up to the right level.	❑	❑	❑	❑	❑
I regard support from my direct superior as important for the quality of my work.	❑	❑	❑	❑	❑
I regard support from the senior insurance physician as important for the quality of my work.	❑	❑	❑	❑	❑
I regard guidance from my direct superior as important for the quality of my work.	❑	❑	❑	❑	❑
I regard professional guidance from the senior insurance physician as important for the quality of my work.	❑	❑	❑	❑	❑
I regard consultation with colleagues as important for the quality of my work.	❑	❑	❑	❑	❑
I regard working in accordance with protocols, guidelines and standards as important for the quality of my work.	❑	❑	❑	❑	❑
I regard good dossier management as important for the quality of my work.	❑	❑	❑	❑	❑
I regard appropriate, achievable production norms as important for the quality of my work.	❑	❑	❑	❑	❑

**Work motivation**					
Please indicate how often each of the following statements applies to you:	Never	Rarely	Sometimes	Often	Always
I regard the work I do as useful and meaningful.	❑	❑	❑	❑	❑
I am enthusiastic about my job.	❑	❑	❑	❑	❑
I find my work inspiring.	❑	❑	❑	❑	❑
I am proud of the work I do.	❑	❑	❑	❑	❑
Please indicate the extent to which you agree with the following statements:	Completely disagree	Disagree to certain extent	Neither agree nor disagree	Agree to certain extent	Completely agree
I have sufficient scope for career development.	❑	❑	❑	❑	❑
I am satisfied with the work I do.	❑	❑	❑	❑	❑
My work suits me.	❑	❑	❑	❑	❑
I enjoy my work.	❑	❑	❑	❑	❑

**Attitude to client**		
Please indicate whether the following principles apply to you.	No	Yes
I believe what the client tells me *in principle*.	❑	❑
It is *in principle* necessary to build up a good relationship with the client during the assessment interview.	❑	❑
In principle, all my assessment interviews follow a fixed pattern.	❑	❑

Please indicate how often each of the following statements applies to you.	Never	Sometimes	Often	Always
I continue the interview until I have collected all the information needed for the assessment.	❑	❑	❑	❑
I treat my clients with respect.	❑	❑	❑	❑
I feel involved with my clients.	❑	❑	❑	❑
I adopt a neutral position, and do not allow myself to be swayed by the interests of the individual client.	❑	❑	❑	❑
I take the time and ask all the necessary questions to build up a good picture of the client's day-to-day situation.	❑	❑	❑	❑
I take the time while writing the report to provide the evidence needed to support my conclusion.	❑	❑	❑	❑
I do not have enough time to write a good report.	❑	❑	❑	❑

**Office standards**					
At our office (in comparison with other offices):	Completely disagree	Disagree to certain extent	Neither agree nor disagree	Agree to certain extent	Completely agree
The main stress is on production.	❑	❑	❑	❑	❑
The main stress is on quality.	❑	❑	❑	❑	❑
Compliance with guidelines and protocols is encouraged.	❑	❑	❑	❑	❑
The aim is on results.	❑	❑	❑	❑	❑

**Environment**				
How important do you consider the opinion of the following persons, groups or bodies to be for the execution of your professional work?	Not important at all	Not very important	Fairly important	Very important
UWV: the senior insurance physician and my direct superior.	❑	❑	❑	❑
UWV: the complaints and appeals committees.	❑	❑	❑	❑
NVVG (the Dutch Association of Insurance Physicians).	❑	❑	❑	❑
UWVA (the association of insurance physicians employed by the UWV).	❑	❑	❑	❑
Family and friends.	❑	❑	❑	❑
Other insurance physicians working at my office.	❑	❑	❑	❑
Other insurance physicians working outside my office.	❑	❑	❑	❑
The public (press and TV).	❑	❑	❑	❑
Professional publications (including PubMed)	❑	❑	❑	❑
Parties responsible for quality testing.	❑	❑	❑	❑
Physicians and other healthcare workers.	❑	❑	❑	❑
The government (the wish to reduce the number of people receiving disability benefit).	❑	❑	❑	❑
Trade unions	❑	❑	❑	❑

**Problems occurring during the assessment interview**				
Please indicate how often the following statements applied to you during the past six months:	Not at all	Sometimes	Often	Always
I always manage to resolve difficult issues involving clients that arise during the assessment interview, if I set myself to it.	❑	❑	❑	❑
I always manage to stick to the proper procedure when a client is being difficult.	❑	❑	❑	❑
It is easy for me to stick to the planned course of the assessment interview and to arrive at the proper assessment.	❑	❑	❑	❑
I am confident that I will be able to find an appropriate response to unexpected events occurring during the assessment interview.	❑	❑	❑	❑
I am inventive enough to be able to find effective ways of coping with unexpected situations during the assessment interview	❑	❑	❑	❑
I can solve most problems that arise during the assessment interview if I set myself to it.	❑	❑	❑	❑
I remain calm when I am faced with problems during the assessment interview, because I am confident that my problem-solving ability will find a way out.	❑	❑	❑	❑
I usually have a number of different solutions if I am faced with a problem during the assessment interview.	❑	❑	❑	❑
I usually know how to respond to an awkward situation I am faced with during the assessment interview.	❑	❑	❑	❑
I always find a way of dealing with the situation, no matter what happens during the assessment interview.	❑	❑	❑	❑

**Task requirements and scope for flexibility**				
Please indicate how often the following statements apply to you in your present position:	Never	Sometimes	Often	Always
I have enough time to do my work properly.	❑	❑	❑	❑
I have to maintain a very high work tempo.	❑	❑	❑	❑
I have a heavy work load.	❑	❑	❑	❑
I have to work extra hard.	❑	❑	❑	❑
My work is monotonous.	❑	❑	❑	❑
My work brings me into emotionally difficult situations.	❑	❑	❑	❑
My work is emotionally demanding.	❑	❑	❑	❑
I get emotionally involved in my work.	❑	❑	❑	❑
I can decide when to perform the assessments.	❑	❑	❑	❑
My working methods are laid down by others.	❑	❑	❑	❑
I determine the sequence in which I carry out my activities.	❑	❑	❑	❑
I decide the approach to be taken to my assessments.	❑	❑	❑	❑
I sometimes have to cut corners in the assessment due to time pressure.	❑	❑	❑	❑

**Fatigue**					
Please indicate how often the following statements apply to you:	Never	A couple of times a year	A couple of times a month	A couple of times a week	Nearly every day
I feel mentally exhausted as a result of my work.	❑	❑	❑	❑	❑
I feel gutted at the end of a working day.	❑	❑	❑	❑	❑
I feel tired when I get up in the morning, with the prospect of another working day ahead of me.	❑	❑	❑	❑	❑
A full day’s work represents a heavy burden for me.	❑	❑	❑	❑	❑
I feel ‘burnt out’ by my work.	❑	❑	❑	❑	❑

Office culture					
Please indicate the extent to which you agree with the following statements:	Completel y disagree	Disagree to certain extent	Neither agree nor disagree	Agree to certain extent	Completel y agree
The level of cooperation at work is good.	❑	❑	❑	❑	❑
We always help one another out at work when necessary.	❑	❑	❑	❑	❑
Conflicts and tensions at work are thoroughly talked through.	❑	❑	❑	❑	❑
We have a common vision of the approach to work at the office.	❑	❑	❑	❑	❑
Consultation at work generally runs smoothly.	❑	❑	❑	❑	❑
We manage to discuss all major issues at work.	❑	❑	❑	❑	❑
All contributions to consultation at work receive appropriate follow-up.	❑	❑	❑	❑	❑
Discussion of practical problems at work often yields solutions.	❑	❑	❑	❑	❑
I have an adequate say on the way in which the workload is shared between the insurance physicians.	❑	❑	❑	❑	❑
The opinion of the insurance physicians is taken sufficiently into account when important decisions are made.	❑	❑	❑	❑	❑
My superior takes any suggestions I have about the work sufficiently seriously.	❑	❑	❑	❑	❑
The opinion of the insurance physicians is taken adequately into account in this organization.	❑	❑	❑	❑	❑

Knowledge and information				
Please indicate how often the following statements apply to you:	Never	Sometime s	Often	Always
I have sufficient medical knowledge to carry out the assessments.	❑	❑	❑	❑
I have sufficient medical information to carry out the assessments.	❑	❑	❑	❑
I get enough information from the occupational physician about attempts to get the client back into the work process.	❑	❑	❑	❑
I get enough information about the diagnosis from the occupational physician.	❑	❑	❑	❑
I get enough feedback from the occupational physician about information from third parties.	❑	❑	❑	❑
If I do not have enough information to perform the assessments, I request it from third parties.	❑	❑	❑	❑
If I do not have enough information I do not request it from third parties, as that would slow down the assessment process too much.	❑	❑	❑	❑
If I have requested supplementary information from third parties, I postpone the assessment until I have got the information.	❑	❑	❑	❑
If I have received supplementary information from third parties, I take that into account in my assessment.	❑	❑	❑	❑
I get sufficient feedback from the Claims manager about the results of my claims assessments.	❑	❑	❑	❑
Information from the RIV (Reintegration report) is decisive for the results of the assessment.	❑	❑	❑	❑

**Techniques** used at start of assessment interview				
Please indicate how often each of the following statements applies to you:	Never	Sometime s	Often	Always
I always take great care to put the client at his or her ease at the start of the interview.	❑	❑	❑	❑
I always take great care to explain the objective of the assessment interview to the client right from the start.	❑	❑	❑	❑
I always take great care to explain the procedure (what points will be discussed, and how) to the client right at the start of the interview.	❑	❑	❑	❑

**Control** over topics dealt with in assessment interview				
Please indicate how often each of the following statements applies to you:	Never	Sometime s	Often	Always
The issues to be discussed will be determined by the client at the start of the interview, and by myself later on.	❑	❑	❑	❑
I always discuss the various issues involved in a fixed order.	❑	❑	❑	❑
If the client brings up a certain point during the interview, I always respond to this.	❑	❑	❑	❑
If the client gives a response that does not really answer the question I asked, I continue that line of questioning until the client understands what I want to know and gives an appropriate answer.	❑	❑	❑	❑
I ask the client to give an example from daily life of every impairment he or she mentions, or which I believe to be present.	❑	❑	❑	❑
Whenever the client mentions an impairment he or she suffers from, I check whether this is likely to lead to activity limitation	❑	❑	❑	❑

**Differences of opinion**					
How do you react to a difference of opinion that occurs between yourself and the client during an interview?	Never	Sometimes	Regularly	Often	Very often
I try to see the client's point of view.	❑	❑	❑	❑	❑
I keep on probing till I find a solution that really satisfies both myself and the client.	❑	❑	❑	❑	❑
I insist on my point of view.	❑	❑	❑	❑	❑
I stress that we will have to find an intermediate solution.	❑	❑	❑	❑	❑
I try to play down the conflict.	❑	❑	❑	❑	❑
I give way to the client's wishes.	❑	❑	❑	❑	❑
I give due weight both to the client's aims and interests and to my own.	❑	❑	❑	❑	❑
I do all I can to persuade the client of my point of view.	❑	❑	❑	❑	❑
I try to reach a compromise wherever possible.	❑	❑	❑	❑	❑
I try to avoid differences of opinion as much as possible.	❑	❑	❑	❑	❑
I adapt my position to suit the client's aims and interests.	❑	❑	❑	❑	❑
I work out a solution that serves my own interests and those of the client as far as possible.	❑	❑	❑	❑	❑
I keep on trying to achieve a result that I am happy with, independent of what the client wants.	❑	❑	❑	❑	❑
I insist that we both have to make compromises.	❑	❑	❑	❑	❑
I try to avoid a confrontation with the client.	❑	❑	❑	❑	❑

Informed consent form

(to be filled in and signed by insurance physician)

This consent form concerns the linkage of your responses to this questionnaire with certain data from the CBBS (*Claimbeoordelings- en Borgingssysteem* = Claim Evaluation & Assessment System). The linkage will be performed by an independent third party, under the supervision of a notary. The notary will check that all data that could be used to identify a person (insurance physician or client) – namely the UWV identification code of the insurance physician and the ID of the office or other organization where the insurance physician is based – will be removed from the file after the linkage has been completed.

Thus, neither the UWV or the researchers will ever be able to identify a person or an office on the basis of the study data.

I hereby give my consent, under the above-mentioned conditions, for the responses I have given to the questionnaire drawn up for the purposes of the study 'Variation in assessment of functional abilities by insurance physicians' to be linked with the CBBS data on the diagnosis, sex and FML (functional capacities list) of the clients assessed by me.
After linkage of the data, the following data will be removed from the file and destroyed.

Surname: ______________________________

First name and initials: ______________________________

Location (UWV office): ______________________________

User id: ______________________________

Date: ______________________________

Signature: ______________________________

THANK YOU FOR TAKING THE TIME TO COMPLETE THIS QUESTIONNAIRE.
